# Association Between Sleep Duration and Obesity Phenotypes in Apparently Healthy Korean Adults: A Cross-Sectional Study Using the Korea National Health and Nutrition Examination Survey 2013–2015

**DOI:** 10.3390/medicina62102014

**Published:** 2026-10-18

**Authors:** Eun Jeong Kim, Jun Hyung Lee, Young Sang Lyu

**Affiliations:** 1 Department of Internal Medicine, Chosun University School of Medicine, Gwangju 61453, Republic of Korea; illusion1211@naver.com (E.J.K.);; 2 Department of Endocrinology and Metabolism, Chosun University Hospital, Chosun University School of Medicine, Gwangju 61453, Republic of Korea

**Keywords:** sleep duration, obesity, metabolically healthy obesity, metabolic syndrome, body size phenotype, KNHANES

## Abstract

*Background and Objectives*: Evidence on the association between sleep duration and obesity phenotypes jointly considering adiposity and metabolic health is limited, particularly in adults without known chronic diseases. We aimed to examine this association in apparently healthy Korean adults. *Materials and Methods*: In this cross-sectional study, we analyzed 7808 apparently healthy adults aged ≥20 years from the Korea National Health and Nutrition Examination Survey 2013–2015, assigned to metabolically healthy normal weight (MHNW), metabolically abnormal normal weight (MANW), metabolically healthy obesity (MHO), or metabolically abnormal obesity (MAO) phenotypes. Self-reported sleep duration was grouped as ≤5, 6, 7, 8, or ≥9 h/day. Survey-weighted multinomial logistic regression was performed with MHNW and ≥9 h as references, adjusting for age and sex (Model 1) and socioeconomic and lifestyle factors (Model 2). Sensitivity analyses used 7 h/day as the reference and a stricter definition of metabolic health; effect modification by sex and age was examined. *Results*: The proportion of MHNW increased from 61.6% (≤5 h) to 76.0% (≥9 h) (*p* < 0.001). In Model 2, sleeping ≤5 h was associated with higher odds of MHO (odds ratio [OR] 1.380, 95% confidence interval [CI] 1.098–1.734) and MAO (OR 1.472, 95% CI 1.109–1.953) relative to 7 h, and these associations remained significant after false discovery rate correction, whereas none of the associations relative to ≥9 h, including that with MANW, did. Under the stricter definition, the association with MAO persisted, whereas that with MHO was no longer statistically significant despite a similar effect estimate (vs. 7 h: OR 1.394, 95% CI 0.926–2.098); no significant interactions by sex or age were observed for MHO or MAO. *Conclusions*: In apparently healthy Korean adults, short sleep duration was associated with higher odds of MHO and MAO, most consistently MAO, but not robustly with MANW. Because of the cross-sectional design, these associations do not imply causality, and prospective studies are needed to determine whether sleep duration is a modifiable target for metabolic health.

## 1. Introduction

Obesity and metabolic syndrome are major and growing public health concerns in Korea. Based on national health screening data, the prevalence of obesity (body mass index [BMI] ≥ 25 kg/m^2^) among Korean adults increased from 30.2% in 2012 to 38.4% in 2021 [1], and the prevalence of metabolic syndrome increased from 23.3% in 2013 to 28.6% in 2022 [2]. At the same time, sleep deprivation is common among Korean adults, and sleep duration decreased further between 2010 and 2022 [3]. Sleep is essential for maintaining metabolic homeostasis, and a growing body of evidence has linked short sleep duration to obesity [4,5], type 2 diabetes [6,7], cardiovascular disease [8], and metabolic syndrome [9,10,11]. Accordingly, sleep health has recently been added as a core component of cardiovascular health in the American Heart Association’s Life’s Essential 8 [12]. Experimental and observational studies suggest several underlying mechanisms, including altered levels of appetite-regulating hormones such as leptin and ghrelin [13,14] and reduced insulin sensitivity [15]; moreover, in randomized studies, experimental sleep restriction increased energy intake and visceral fat accumulation [16], whereas sleep extension reduced energy intake in adults with overweight [17]. Furthermore, the relationship between sleep duration and adverse outcomes is often U-shaped, with risks elevated at both extremes [6,8,11]; hence, the selection of the reference category is methodologically important.

Obesity, however, is a heterogeneous condition, and BMI alone does not fully capture cardiometabolic risk. To capture this heterogeneity, individuals can be cross-classified by both adiposity and metabolic status into four groups, namely metabolically healthy normal weight (MHNW), metabolically abnormal normal weight (MANW), metabolically healthy obesity (MHO), and metabolically abnormal obesity (MAO) [18,19,20], although the cardiometabolic risk attributed to MHO depends considerably on how metabolic health is defined [20]. Normal-weight individuals with metabolic abnormalities may have insulin resistance and visceral adiposity, and thus a cardiometabolic risk that is not reflected by body weight alone [21,22]. This issue is especially important for Asians, in whom metabolic derangements often emerge at a lower BMI than in Western populations [23].

Although the relationship between sleep duration and obesity or metabolic syndrome has been examined extensively, less is known about how sleep duration relates to obesity phenotypes that jointly consider adiposity and metabolic health. Previous KNHANES-based studies have examined sleep duration in relation to metabolic syndrome [10], and an analysis of the KNHANES 2010–2012 showed that obese phenotypes had a shorter mean sleep duration than non-obese phenotypes [24]; however, in the latter study, sleep duration was examined as an outcome rather than as a categorized exposure. Treating sleep duration as the exposure allows the odds of each phenotype to be estimated across sleep-duration categories with adjustment for confounders and makes it possible to evaluate graded or threshold patterns and the sensitivity of the findings to the choice of the reference category, which cannot be addressed by comparing mean sleep duration between phenotypes. In Chinese young workers, sleeping < 7 h was associated with higher odds of metabolically healthy and unhealthy overweight/obesity, but not of the metabolically unhealthy normal-weight phenotype [25]; however, that study was limited to a young occupational population. In particular, evidence in adults without known chronic diseases is limited; in this population, associations are less likely to be influenced by disease-related sleep disturbances or medication use. Therefore, the objective of this study was to examine the association between self-reported sleep duration and obesity phenotypes (MHNW, MANW, MHO, and MAO) in apparently healthy Korean adults using data from the Korea National Health and Nutrition Examination Survey (KNHANES) 2013–2015. We hypothesized that short sleep duration would be associated with higher odds of the MANW, MHO, and MAO phenotypes.

## 2. Materials and Methods

### 2.1. Study Population

We analyzed the 2013–2015 cycles of the KNHANES, administered by the Korea Disease Control and Prevention Agency (KDCA). The KNHANES is a continuing nationwide survey with a cross-sectional design; a representative sample of the non-institutionalized Korean population is drawn each year by stratified, multistage, clustered probability sampling, and trained staff administer its health interview, nutrition, and health examination components [26]. The sixth KNHANES (2013–2015) included 22,948 participants, with a response rate of 78.3% [27]. We used the 2013–2015 data because sleep duration was assessed using a single question on average daily sleep duration in these survey years, whereas from 2016 onward, sleep was assessed using separate bedtimes and wake-up times on weekdays and weekends, and these items were administered only intermittently (2016–2018 and 2021) [28]. Using the 2013–2015 data therefore allowed a consistent sleep measure across the three survey years and comparability with previous KNHANES-based studies [24], and avoided the influence of changes in sleep and lifestyle during the COVID-19 pandemic on more recent data [3,28].

**Figure 1:**
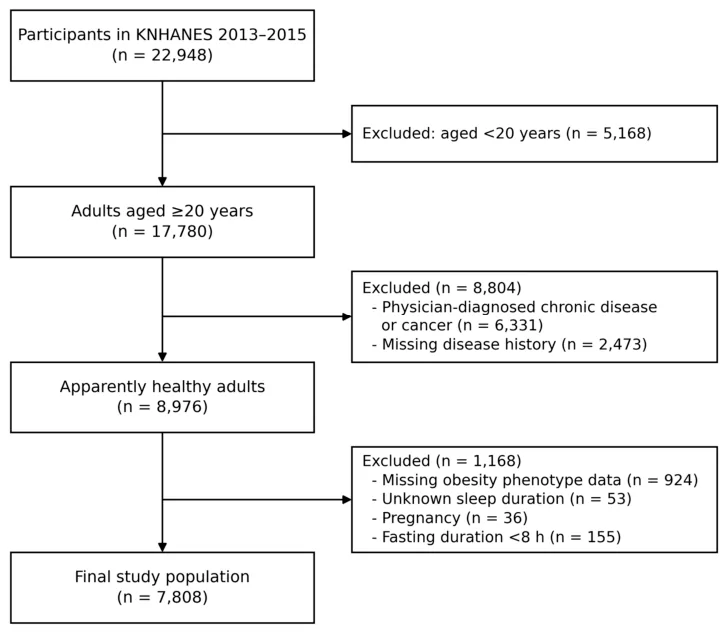
Flow diagram of participant selection. KNHANES, Korea National Health and Nutrition Examination Survey.

Of the 22,948 individuals who participated in the KNHANES 2013–2015, we excluded those aged <20 years (n = 5168). Following the definition used in our previous study [29], apparently healthy adults were identified by excluding participants with a physician diagnosis of hypertension, dyslipidemia, diabetes mellitus, stroke, myocardial infarction, angina pectoris, pulmonary tuberculosis, asthma, thyroid disease, renal failure, liver cirrhosis, or cancer (n = 6331) and those with missing information on disease history (n = 2473). Among the remaining 8976 participants, we further excluded those with missing data required to define obesity phenotypes (n = 924), those who did not report their sleep duration (n = 53), pregnant women (n = 36), and those who had fasted for <8 h before blood sampling (n = 155). Finally, 7808 apparently healthy adults were included in the analysis (Figure 1).

The KNHANES protocol received approval from the KDCA Institutional Review Board, and written informed consent was obtained from every participant. Ethical approval for this secondary analysis was granted by the Institutional Review Board of Chosun University Hospital (approval number: 2024-09-0260), and the study adhered to the principles of the Declaration of Helsinki.

### 2.2. Assessment of Sleep Duration

Participants reported their usual daily sleep duration in response to a single questionnaire item, and the responses were grouped into five categories (≤5, 6, 7, 8, and ≥9 h/day). Information on sleep quality, napping, and sleep disorders such as obstructive sleep apnea was not collected in the KNHANES 2013–2015.

### 2.3. Anthropometric and Laboratory Measurements

Trained examiners performed the anthropometric measurements according to standardized protocols. BMI was computed as body weight (kg) divided by height squared (m^2^). Waist circumference (WC) was measured halfway between the lowest rib and the top of the iliac crest. Three blood pressure readings were taken with a standard mercury sphygmomanometer while participants were seated after resting for ≥5 min, and the mean of the last two readings was used. Blood was drawn after an overnight fast of ≥8 h. Serum total cholesterol, triglycerides (TG), high-density lipoprotein cholesterol (HDL-C), and fasting plasma glucose (FPG) levels were determined with an automated analyzer (Hitachi Automatic Analyzer 7600-210, Hitachi, Tokyo, Japan), and glycated hemoglobin (HbA1c) was determined by high-performance liquid chromatography (Tosoh G8, Tosoh Bioscience, Tokyo, Japan). Low-density lipoprotein cholesterol (LDL-C) was measured directly when available; otherwise, it was calculated using the Friedewald equation in participants with TG < 400 mg/dL.

### 2.4. Definition of Obesity Phenotypes

Metabolic syndrome was defined as the presence of at least three of the following five components: (1) central obesity, using the Korean Society for the Study of Obesity cut-offs for WC (men, ≥90 cm; women, ≥85 cm) [30]; (2) hypertriglyceridemia (TG ≥ 150 mg/dL) or treatment for elevated TG; (3) low HDL-C (<40 mg/dL in men or <50 mg/dL in women) or use of lipid-lowering medication; (4) elevated blood pressure (systolic ≥ 130 mmHg and/or diastolic ≥ 85 mmHg) or use of antihypertensive medication; and (5) impaired fasting glucose (FPG ≥ 100 mg/dL) or use of glucose-lowering agents. Because participants with a physician diagnosis of hypertension, dyslipidemia, or diabetes were excluded, no participant met any component solely on the basis of medication use; thus, metabolic syndrome status was determined entirely by measured values.

Participants were then classified into four obesity phenotypes according to BMI, using the Asian-specific cut-off of 25 kg/m^2^ [23], and the presence or absence of metabolic syndrome. Participants with a BMI < 25 kg/m^2^ were classified as MHNW or MANW, and those with a BMI ≥ 25 kg/m^2^ as MHO or MAO, depending on whether metabolic syndrome was absent or present, respectively. Underweight participants (BMI < 18.5 kg/m^2^) were included in the normal-weight categories. In a sensitivity analysis, a stricter definition of metabolic health was applied, in which participants were classified as metabolically healthy only if they had none of the four metabolic syndrome components other than WC (elevated TG, low HDL-C, elevated blood pressure, and elevated FPG), because WC is closely correlated with BMI [20,25]. In another sensitivity analysis, underweight participants (n = 401) were excluded.

### 2.5. Covariates

Information on household income, educational attainment, residential area, smoking status, alcohol drinking, physical activity, perceived stress, work schedule, and dietary intake was obtained through health interviews, self-administered questionnaires, and the nutrition survey. Household income was categorized into quartiles, and educational attainment was categorized as middle school or less, high school, or college or higher. Smoking status was classified as never, past, or current smoking. Participants who reported any alcohol consumption during the previous year were classified as drinkers. Regular exercise referred to ≥150 min/week of moderate-intensity or ≥75 min/week of vigorous-intensity physical activity. Perceived stress was dichotomized as high (feeling “very much” or “quite a lot” of stress in daily life) or low, and total energy intake (kcal/day) was estimated from the 24 h dietary recall. Work schedule was classified as day work (usually working between 6 a.m. and 6 p.m.), non-day work (evening, night, regular rotating, 24 h rotating, split, irregular rotating, or other shifts), or not working during the past year.

Covariates for the main multivariable model were selected a priori on the basis of previous evidence and a conceptual framework of confounding, rather than by statistical selection procedures [31]. We included factors that are plausibly associated with both sleep duration and metabolic status—age, sex, household income, educational attainment, smoking status, alcohol drinking, and regular exercise—and that are unlikely to lie on the causal pathway between sleep duration and obesity phenotypes. Total energy intake was not included in the main model because it may act as a mediator of the association between short sleep and adiposity [16,17]; instead, it was considered in an additional model together with residential area, perceived stress, and work schedule.

### 2.6. Statistical Analysis

Following the KDCA analytic guidelines, the complex sampling design of the KNHANES (strata, primary sampling units, and sampling weights) was incorporated into every analysis; integrated weights for the 3-year period were calculated by dividing the health interview and examination weights by 3. Variances were estimated using Taylor series linearization, with the analytic sample treated as a subpopulation. Weighted means ± standard errors (SE) are reported for continuous variables, and unweighted numbers with weighted percentages for categorical variables. Differences according to sleep duration were tested using design-adjusted Wald F tests.

Survey-weighted multinomial logistic regression was used to estimate the association of sleep duration with obesity phenotypes, with obesity phenotype as the dependent variable (MHNW as the reference category) and sleep duration as the independent variable (≥9 h as the reference category). Model 1 included age and sex as covariates; Model 2 additionally included household income, educational attainment, smoking status, alcohol drinking, and regular exercise; and Model 3 additionally included residential area, perceived stress, total energy intake, and work schedule. Because the choice of the reference category may influence the estimates, a sensitivity analysis was performed in which the multinomial logistic regression analyses (Models 1 and 2) were repeated using 7 h/day, the most common sleep duration in the study population, as the reference category. Additional sensitivity analyses were performed using the stricter definition of metabolic health and after excluding underweight participants. To examine effect modification, analyses were stratified by sex and age group (20–39 and ≥40 years), and interaction terms between sleep duration and sex or age group were tested jointly and separately for each phenotype using design-adjusted Wald F tests. Because the number of participants sleeping ≥9 h was small within strata, 7 h was used as the reference category in the stratified analyses.

Multicollinearity among covariates was assessed using variance inflation factors (VIFs), and all VIFs were below 5 (maximum, 4.18 for the sleep-duration indicator variables), with all generalized VIFs adjusted for degrees of freedom ≤1.41. The overall significance of each model was assessed using design-adjusted Wald F tests; because formal goodness-of-fit tests for survey-weighted multinomial models are not well established, model adequacy was further examined by comparing the estimates across nested models and sensitivity analyses. Multivariable analyses were performed as complete-case analyses: of the 7808 participants, 62 (0.8%) had missing data on at least one covariate in Model 2 (household income, n = 33; educational attainment, n = 13; smoking status, n = 1; regular exercise, n = 24) and were excluded from Model 2. The excluded participants were older (weighted mean age, 47.8 vs. 40.5 years), but the proportion of MHNW was similar (68.0% vs. 68.0%). Given the small proportion of missing data, multiple imputation was not performed [32]. No formal adjustment for multiple comparisons was applied in the primary analyses because the analyses addressed a prespecified hypothesis and the estimates across sleep-duration categories and phenotypes were correlated [33]; therefore, results with *p* values close to 0.05 were interpreted with caution, and we focused on the consistency of the associations across models and sensitivity analyses rather than on individual *p* values. As a supplementary check, the Benjamini–Hochberg false discovery rate (FDR) procedure was applied to the unrounded *p* values of the 12 sleep-duration-by-phenotype comparisons in Model 2 for each reference category. ORs and 95% CIs are presented, and a two-sided *p* value < 0.05 was considered statistically significant. SPSS version 32.0 (IBM Corp., Armonk, NY, USA) was used for the statistical analyses; the additional sensitivity, interaction, and FDR analyses were performed using Python version 3.13 with the same Taylor series linearization variance estimation, which reproduced the SPSS estimates of the main models.

## 3. Results

### 3.1. Participant Characteristics by Sleep Duration

The analytic sample comprised 7808 apparently healthy adults. The weighted distribution of sleep duration was 12.7% for ≤5 h, 28.9% for 6 h, 30.4% for 7 h, 21.5% for 8 h, and 6.5% for ≥9 h (Table 1). Mean age decreased with longer sleep duration, from 43.6 years in the ≤5 h group to 35.5 years in the ≥9 h group. The proportion of men was lowest in the ≥9 h group (43.0%). Socioeconomic factors showed a U-shaped pattern: the proportion of participants in the lowest household income quartile was highest in the ≥9 h (17.4%) and ≤5 h (13.6%) groups, and the proportion with college or higher education was lowest in these two groups (35.4% and 36.7%, respectively). The proportion of current smokers was highest in the ≤5 h group (29.9%), and alcohol drinking was least common in the ≤5 h and ≥9 h groups.

BMI (23.8 kg/m^2^ in the ≤5 h group vs. 22.6 kg/m^2^ in the ≥9 h group), WC (81.0 vs. 77.8 cm), systolic blood pressure (115.3 vs. 110.0 mmHg), and diastolic blood pressure (75.4 vs. 71.5 mmHg) were higher among those with shorter sleep duration (all *p* < 0.001). HbA1c, total cholesterol, and LDL-C also differed significantly according to sleep duration, although the absolute differences were small, whereas FPG, HDL-C, and TG did not differ significantly. The prevalence of metabolic syndrome decreased from 19.4% in the ≤5 h group to 10.8% in the ≥9 h group (*p* < 0.001).

**Figure 2:**
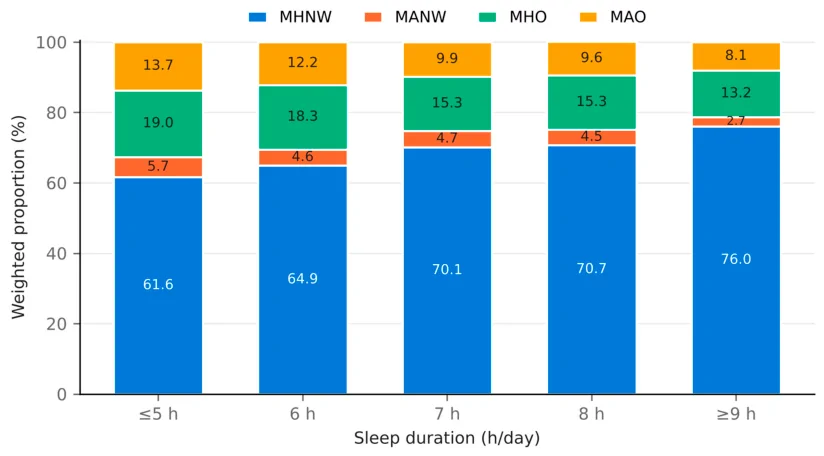
Distribution of obesity phenotypes according to sleep duration. Values are weighted percentages. MANW, metabolically abnormal normal weight; MAO, metabolically abnormal obesity; MHNW, metabolically healthy normal weight; MHO, metabolically healthy obesity.

Obesity phenotype distribution varied significantly across sleep-duration categories (*p* < 0.001; Figure 2). The proportion of MHNW increased progressively with longer sleep duration, from 61.6% in the ≤5 h group to 76.0% in the ≥9 h group. Conversely, the proportions of MAO (13.7% vs. 8.1%), MHO (19.0% vs. 13.2%), and MANW (5.7% vs. 2.7%) were higher in the ≤5 h group than in the ≥9 h group. Overall, the non-MHNW phenotypes (MANW, MHO, or MAO) accounted for 38.4% of those sleeping ≤5 h and 24.0% of those sleeping ≥9 h.

### 3.2. Participant Characteristics by Obesity Phenotype

Participant characteristics by obesity phenotype are summarized in Table 2. The weighted prevalence was 68.0% for MHNW, 4.6% for MANW, 16.5% for MHO, and 10.9% for MAO. Participants with MANW were the oldest (51.4 years) and had the lowest educational attainment, whereas those with MHO were similar in age to those with MHNW (39.8 vs. 39.4 years). The proportion of men and current smokers was higher in the other three phenotypes than in the MHNW phenotype. As expected, compared with the MHNW phenotype, the MANW and MAO phenotypes had higher BMI, WC, blood pressure, FPG, HbA1c, and TG and lower HDL-C. Sleep duration also differed according to obesity phenotype (*p* < 0.001). Mean sleep duration was longest in the MHNW phenotype (6.87 h/day) and shorter in the MANW (6.64 h/day), MHO (6.67 h/day), and MAO (6.64 h/day) phenotypes. The proportion of participants sleeping ≤6 h was 39.0% in the MHNW phenotype, compared with 44.4%, 46.8%, and 48.6% in the MANW, MHO, and MAO phenotypes, respectively, whereas the proportion sleeping ≥9 h was highest in the MHNW phenotype (7.2%).

### 3.3. Sleep Duration and Risk of Obesity Phenotypes

**Figure 3:**
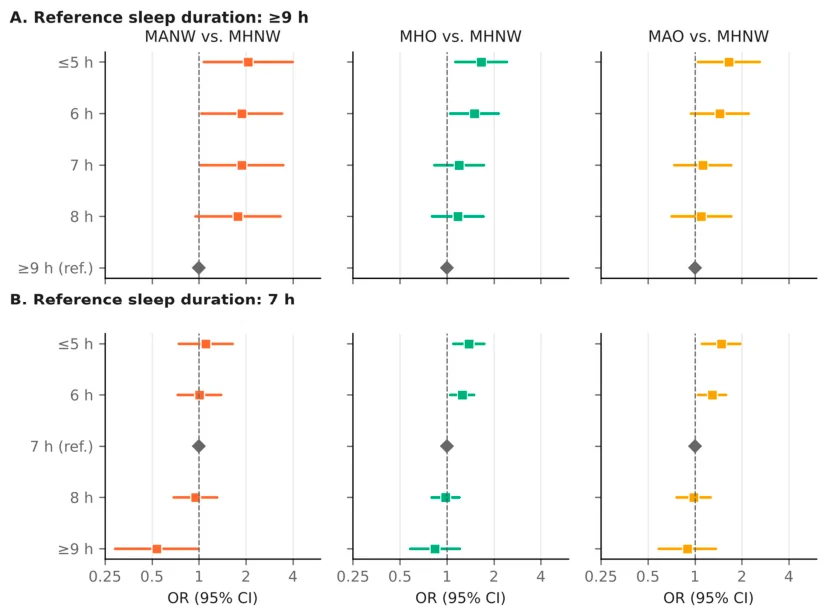
Association between sleep duration and obesity phenotypes in Model 2. Squares indicate ORs and horizontal lines indicate 95% CIs, estimated using survey-weighted multinomial logistic regression with MHNW as the reference phenotype and (**A**) ≥9 h or (**B**) 7 h as the reference sleep duration (diamonds), adjusted for age, sex, household income, educational attainment, smoking status, alcohol drinking, and regular exercise. The *x*-axis is on a logarithmic scale. CI, confidence interval; MANW, metabolically abnormal normal weight; MAO, metabolically abnormal obesity; MHNW, metabolically healthy normal weight; MHO, metabolically healthy obesity; OR, odds ratio.

Table 3 and Figure 3 present the multinomial logistic regression results. In Model 1, participants who slept ≤5 h had significantly higher odds of MANW (OR 1.933, 95% CI 1.033–3.619), MHO (OR 1.707, 95% CI 1.173–2.484), and MAO (OR 1.696, 95% CI 1.078–2.669) than those who slept ≥9 h. Sleeping 6 h was also associated with higher odds of MHO (OR 1.493, 95% CI 1.057–2.109), whereas the ORs for 7 h and 8 h of sleep were not statistically significant for any phenotype.

After further adjustment for socioeconomic and lifestyle factors (Model 2, n = 7746), the associations for the ≤5 h group remained significant for MANW (OR 2.063, 95% CI 1.070–3.978), MHO (OR 1.650, 95% CI 1.128–2.413), and MAO (OR 1.649, 95% CI 1.047–2.597). Sleeping 6 h was associated with higher odds of MANW (OR 1.876, 95% CI 1.033–3.409) and MHO (OR 1.495, 95% CI 1.049–2.131), and sleeping 7 h was associated with higher odds of MANW (OR 1.870, 95% CI 1.010–3.460). For MHO and MAO, the ORs showed a graded decrease from the ≤5 h group to the 8 h group, whereas for MANW the ORs were similar across all categories below 9 h, with wide CIs (Figure 3A).

In the sensitivity analysis using 7 h as the reference category (Table S1 and Figure 3B), the associations of short sleep with MHO and MAO were consistent with those of the main analysis. In Model 2, sleeping ≤5 h was associated with higher odds of MHO (OR 1.380, 95% CI 1.098–1.734) and MAO (OR 1.472, 95% CI 1.109–1.953), and sleeping 6 h was also associated with higher odds of MHO (OR 1.251, 95% CI 1.048–1.492) and MAO (OR 1.287, 95% CI 1.047–1.583). In contrast, sleeping 8 h or ≥9 h was not associated with MHO or MAO. For MANW, the ORs for ≤5 h (1.104, 95% CI 0.742–1.641) and 6 h (1.003, 95% CI 0.729–1.381) were not significant, whereas sleeping ≥9 h was associated with lower odds of MANW (OR 0.535, 95% CI 0.289–0.990). The results of Model 1 were similar (Table S1).

### 3.4. Additional Sensitivity and Subgroup Analyses

When the stricter definition of metabolic health was applied (Table S2), the weighted prevalence of MHO decreased to 5.9% (n = 433) and that of MAO increased to 21.5%. Sleeping ≤ 5 h remained associated with higher odds of MAO compared with sleeping ≥ 9 h (OR 1.675, 95% CI 1.146–2.447) and 7 h (OR 1.395, 95% CI 1.099–1.771) in Model 2. The ORs for MHO were of similar magnitude to those in the main analysis but were not statistically significant (vs. ≥9 h: OR 1.397, 95% CI 0.805–2.423; vs. 7 h: OR 1.394, 95% CI 0.926–2.098), and no association was observed for MANW. After excluding underweight participants (n = 401; Table S3), the associations of sleeping ≤ 5 h with MHO and MAO relative to 7 h remained significant (OR 1.363, 95% CI 1.084–1.713, and OR 1.464, 95% CI 1.103–1.944, respectively), whereas relative to ≥9 h the association with MAO was attenuated (OR 1.514, 95% CI 0.964–2.378). In Model 3, further adjusted for residential area, perceived stress, total energy intake, and work schedule (n = 6854; Table S4), sleeping ≤ 5 h remained associated with MHO and MAO relative to 7 h (OR 1.388, 95% CI 1.086–1.775, and OR 1.426, 95% CI 1.043–1.949, respectively), and the estimates were nearly identical to those of Model 2 fitted in the same participants (OR 1.406 and 1.455, respectively).

In the stratified analyses (Table S5), the ORs of sleeping ≤ 5 h for MHO and MAO were above 1 in all subgroups (range, 1.233–1.641), although not all were statistically significant owing to the smaller sample sizes. No significant interaction was observed between sleep duration and sex (overall *p* for interaction = 0.119; MHO, *p* = 0.983; MAO, *p* = 0.547) or age group (overall *p* for interaction = 0.870; MHO, *p* = 0.230; MAO, *p* = 0.890). For MANW, a significant interaction with sex was observed (*p* = 0.006), driven mainly by lower odds of MANW among men sleeping ≥9 h. After FDR correction, the associations of sleeping ≤ 5 h with MHO and MAO relative to 7 h remained significant (*q* = 0.045) and those of sleeping 6 h were of borderline significance (*q* = 0.050), whereas none of the associations relative to ≥9 h remained significant (all *q* ≥ 0.093). The VIFs for the covariates are presented in Table S6.

## 4. Discussion

In this nationally representative sample of apparently healthy Korean adults, those sleeping ≤ 5 h had higher odds of MHO and MAO than those sleeping ≥ 9 h, and these associations persisted after adjustment for socioeconomic and lifestyle factors and were consistent when 7 h was used as the reference category. After FDR correction, however, only the associations relative to 7 h remained significant. An association with MANW was also observed in the main analysis, but it was not robust to the choice of the reference category. The MANW, MHO, and MAO phenotypes together accounted for 38.4% of those sleeping ≤ 5 h compared with 24.0% of those sleeping ≥ 9 h. These findings are in line with previous studies linking short sleep to obesity [4,5], metabolic syndrome [9,10,11], and type 2 diabetes [6,7]. They are also consistent with an earlier KNHANES analysis showing shorter sleep duration in obese phenotypes [24] and with a study of Chinese young workers in which short sleep was associated with metabolically healthy and unhealthy overweight/obesity, but not with the metabolically unhealthy normal-weight phenotype [25]. Our study extends these findings by showing that short sleep is associated with obesity phenotypes irrespective of metabolic health, even in adults without known chronic diseases. Unlike the earlier KNHANES analysis, which compared mean sleep duration across phenotypes [24], we examined sleep duration as a categorized exposure; this allowed us to estimate the odds of each phenotype across the full range of sleep duration, to identify a threshold-type pattern below 7 h, and to assess the robustness of the associations to the choice of the reference category and to correction for multiple comparisons.

Several mechanisms may explain the association between short sleep and the MHO and MAO phenotypes. Experimental sleep restriction lowers leptin and raises ghrelin concentrations, thereby stimulating hunger and appetite [13,14], and impairs glucose tolerance and insulin sensitivity [15,34]. In a randomized crossover study, experimental sleep restriction increased energy intake and visceral fat accumulation [16], and in a randomized clinical trial, sleep extension reduced energy intake in adults with overweight in a real-life setting [17]. Sleep loss is also associated with elevated evening cortisol levels [35] and low-grade systemic inflammation [36], and short sleep duration has been associated with abdominal fat accumulation [37]. These pathways may contribute to insulin resistance and visceral adiposity, which characterize both MAO and MANW [21,22]. In addition, short sleepers in our study had lower household income and educational attainment and a higher prevalence of current smoking; however, the associations remained significant after adjustment for these factors, suggesting that they do not fully explain the observed relationship. Conversely, obesity and metabolic abnormalities may themselves shorten or fragment sleep, for example through sleep-disordered breathing, and this bidirectional relationship cannot be disentangled in a cross-sectional study.

The MANW phenotype deserves particular attention because it is easily overlooked in clinical practice. In the main analysis, the ORs for MANW were elevated in all groups sleeping less than 9 h. However, no clear dose–response gradient was observed; the CIs were wide, and the associations were not significant when 7 h was used as the reference category. Instead, the sensitivity analysis showed that long sleep (≥9 h) was associated with lower odds of MANW relative to 7 h of sleep, suggesting that the associations observed in the main analysis were largely driven by the low prevalence of MANW in the small ≥9 h reference group (2.7%). Therefore, our findings do not provide robust evidence of an association between short sleep and MANW, and further studies with larger numbers of individuals with MANW are needed.

Short sleep was also associated with MHO, which is often regarded as a relatively benign or transitional state [18,19]. The ORs for MHO and MAO decreased in a graded manner with longer sleep duration, and the associations for MHO were significant in both the ≤5 h and 6 h groups, regardless of whether ≥9 h or 7 h was used as the reference category. Because MHO defined by the absence of metabolic syndrome allows up to two metabolic abnormalities, including elevated WC, and because the cardiometabolic risk of MHO depends substantially on the definition used [20,38], we additionally applied a stricter definition that allowed no metabolic abnormality other than elevated WC. Under this definition, the association with MAO persisted, whereas the ORs for MHO were similar in magnitude to those in the main analysis but were no longer statistically significant, with wide CIs reflecting the small size of the strictly defined MHO group (5.9%). Therefore, part of the association between short sleep and MHO in the main analysis may reflect individuals with one or two metabolic abnormalities, and this association should be interpreted with caution. Because a substantial proportion of individuals with MHO progress to metabolically unhealthy obesity over time [19], lifestyle factors associated with MHO, including short sleep, may warrant investigation in longitudinal and interventional studies to determine whether they contribute to this transition.

The association between sleep duration and obesity may differ by sex and age. A recent meta-analysis found that short sleep was associated with incident obesity in both men and women, without a significant difference between the sexes [5], whereas a meta-analysis of metabolic syndrome found no association between short sleep and metabolic syndrome in adults aged ≥65 years [11]. In our study, the ORs of short sleep for MHO and MAO were above 1 in all sex and age subgroups, and no significant interaction was observed for these phenotypes, although the associations were somewhat weaker in women and the stratified analyses had limited statistical power. The significant sex interaction for MANW was driven by lower odds of MANW among men sleeping ≥ 9 h, which further suggests that the MANW findings are unstable.

The choice of the reference category warrants consideration. In the main analysis, we used ≥9 h of sleep as the reference to contrast short sleepers with those at the opposite end of the sleep-duration distribution. However, long sleep duration has also been associated with adverse health outcomes in a U-shaped manner [6,8,11], and in our study the ≥9 h group was the smallest (6.5%), the youngest, and had the lowest socioeconomic status. Although age was included in all models and socioeconomic factors were added in Model 2, residual confounding related to these characteristics cannot be excluded, and the small size of this group resulted in wide CIs. To address these concerns, we performed a sensitivity analysis using 7 h, the most common sleep duration in our study population and within the range generally recommended for adults, as the reference category. The associations of short sleep (≤6 h) with MHO and MAO were consistent in this analysis, and 8 h or ≥9 h of sleep was not associated with these phenotypes, indicating a threshold-type association in which the odds increased mainly below 7 h of sleep. These findings support the robustness of the associations with MHO and MAO, whereas the association with MANW should be interpreted with caution. Moreover, although no formal adjustment for multiple comparisons was applied in the primary analysis, the associations of ≤5 h of sleep with MHO and MAO relative to 7 h remained significant after FDR correction, whereas none of the associations relative to ≥9 h, including those for MANW, did; therefore, individual associations with *p* values close to 0.05, including those for 6 h of sleep, should be interpreted cautiously, and our conclusions are based primarily on the comparisons with 7 h as the reference.

From a clinical perspective, our findings indicate that short sleep duration frequently coexists with the MHO and MAO phenotypes, even in adults without diagnosed chronic diseases, and that sleep habits may be worth considering when lifestyle factors are evaluated in individuals with obesity. However, because of the cross-sectional design, the observed associations do not indicate that short sleep causes these phenotypes or that extending sleep would improve metabolic health; reverse causation and residual confounding remain plausible explanations. Whether sleep duration is a modifiable target for the prevention of unfavorable obesity phenotypes therefore needs to be tested in prospective and interventional studies.

This study has several strengths. First, we used data from a large sample representative of Korean adults and accounted for the complex survey design throughout the analyses. Second, obesity phenotypes were defined by jointly considering adiposity and metabolic health, which allowed a more nuanced assessment than BMI alone. Third, restricting the analysis to apparently healthy adults, defined by the exclusion of participants with clinically diagnosed chronic diseases [29], reduced the influence of overt disease and its treatment on sleep and metabolic status. Fourth, the associations with MHO and MAO were consistent after adjustment for socioeconomic and lifestyle factors and across several sensitivity analyses.

Nevertheless, some limitations should be acknowledged. First, because of its cross-sectional design, causality cannot be inferred, and reverse causation is possible; for example, obesity and metabolic abnormalities may lead to shorter or poorer sleep through sleep-disordered breathing or other mechanisms. Therefore, our findings should not be interpreted as evidence that sleep duration is a modifiable cause of unfavorable obesity phenotypes. Second, sleep duration was self-reported using a single question and assessed as average daily sleep duration; sleep quality, napping, weekday–weekend differences, and sleep disorders such as obstructive sleep apnea were not assessed, and self-reported sleep duration may differ from objectively measured sleep. Such misclassification may have biased the observed associations; if it was non-differential with respect to obesity phenotype, it would most likely have attenuated them, but differential misreporting according to body weight or metabolic status cannot be excluded. Third, although we adjusted for major socioeconomic and lifestyle factors, and additionally for perceived stress, total energy intake, and work schedule in a sensitivity analysis, residual confounding by unmeasured or imprecisely measured factors, such as diet quality, depressive symptoms, and the duration of shift work, cannot be excluded; moreover, a further 892 participants were excluded from Model 3, mainly because of missing dietary data, which may have introduced selection bias, although the estimates of Model 2 fitted in the same participants were nearly identical. Because factors such as depressive symptoms and poor diet quality are associated with both short sleep and adverse metabolic profiles, residual confounding may have inflated the observed associations, and their magnitude should be interpreted with caution. Fourth, metabolic health was defined using metabolic syndrome criteria, and direct measures of insulin resistance, body fat distribution, or inflammation were not available. In addition, underweight participants were included in the normal-weight categories, which may have introduced heterogeneity into these groups, although the associations relative to 7 h were similar after excluding them; furthermore, the association with MHO was no longer statistically significant despite a similar effect estimate under a stricter definition of metabolic health. Fifth, the ≥9 h reference group was small (6.5%) and differed from the other groups in age and socioeconomic characteristics, and the associations relative to this group did not remain significant after correction for multiple comparisons. Sixth, the data were collected in 2013–2015, and the prevalence of obesity, metabolic syndrome, and short sleep in Korea has changed since then [1,2,3]; therefore, the prevalence estimates may not reflect the current population, and replication in more recent data with updated sleep measures is warranted. Finally, “apparently healthy” status was defined by the absence of clinically diagnosed chronic diseases; undiagnosed conditions, such as undiagnosed hypertension, diabetes, or sleep apnea, cannot be excluded, and the exclusion of participants with diagnosed conditions such as hypertension, dyslipidemia, and diabetes, who are more likely to have the MANW or MAO phenotypes, may have led to an underestimation of the prevalence of these phenotypes and limits the generalizability of our findings to the general population and to other ethnic populations.

## 5. Conclusions

Among apparently healthy Korean adults, sleeping ≤5 h per day was linked to higher odds of the MHO and MAO phenotypes, after adjustment for socioeconomic and lifestyle factors, and these associations relative to 7 h of sleep remained significant after FDR correction. By contrast, none of the associations relative to ≥9 h remained significant after FDR correction, and these should be interpreted cautiously. The association with MAO was also robust to a stricter definition of metabolic health, whereas the association with MHO was no longer statistically significant under the stricter definition, despite a similar effect estimate. The association with MANW was also sensitive to the choice of the reference category and should not be regarded as established. Because of the cross-sectional design, these findings reflect associations and do not establish that short sleep causes unfavorable obesity phenotypes. Prospective and interventional studies are needed to determine whether sleep duration is a modifiable factor and whether improving sleep duration can prevent the development of metabolically unhealthy phenotypes and obesity.

## Figures and Tables

**Table 1:** Participant characteristics by sleep duration category.

	≤5 h	6 h	7 h	8 h	≥9 h	*p* Value
n (weighted %)	1036 (12.7)	2271 (28.9)	2347 (30.4)	1660 (21.5)	494 (6.5)	
Age (years)	43.6 ± 0.5	41.4 ± 0.3	40.3 ± 0.3	39.4 ± 0.4	35.5 ± 0.8	<0.001
Male sex (%)	48.4	55.6	51.2	50.4	43.0	<0.001
Household income (%)						<0.001
Quartile 1 (lowest)	13.6	8.4	7.1	8.7	17.4	
Quartile 2	23.3	23.7	23.9	25.6	26.5	
Quartile 3	31.7	31.0	33.1	34.1	30.2	
Quartile 4 (highest)	31.5	36.9	36.0	31.6	25.9	
Education (%)						<0.001
Middle school or less	23.7	12.8	12.2	13.7	16.8	
High school	39.7	40.9	38.6	43.5	47.8	
College or higher	36.7	46.3	49.2	42.8	35.4	
Urban residence (%)	84.8	84.4	86.4	84.6	79.8	0.045
Smoking status (%)						0.020
Never	54.4	53.9	56.4	56.6	60.3	
Past	15.7	19.4	19.3	17.4	15.4	
Current	29.9	26.7	24.3	26.0	24.3	
Alcohol drinking (%)	76.0	82.1	83.8	82.0	76.3	<0.001
Regular exercise ^a^ (%)	53.4	53.4	52.8	51.8	47.5	0.308
BMI (kg/m^2^)	23.8 ± 0.1	23.7 ± 0.1	23.2 ± 0.1	23.1 ± 0.1	22.6 ± 0.3	<0.001
WC (cm)	81.0 ± 0.4	80.5 ± 0.2	79.0 ± 0.2	78.9 ± 0.3	77.8 ± 0.6	<0.001
SBP (mmHg)	115.3 ± 0.6	113.5 ± 0.4	112.9 ± 0.4	112.7 ± 0.4	110.0 ± 0.7	<0.001
DBP (mmHg)	75.4 ± 0.4	75.3 ± 0.3	74.5 ± 0.3	74.4 ± 0.3	71.5 ± 0.5	<0.001
FPG (mg/dL)	94.7 ± 0.5	95.0 ± 0.4	93.9 ± 0.3	93.9 ± 0.3	93.1 ± 0.9	0.050
HbA1c (%)	5.57 ± 0.02	5.56 ± 0.01	5.52 ± 0.01	5.51 ± 0.01	5.50 ± 0.03	0.005
Total cholesterol (mg/dL)	188.5 ± 1.2	189.9 ± 0.8	187.6 ± 0.8	186.2 ± 1.0	181.5 ± 2.0	<0.001
LDL-C (mg/dL)	113.1 ± 1.1	114.5 ± 0.7	112.4 ± 0.7	111.5 ± 0.9	107.9 ± 1.7	0.002
HDL-C (mg/dL)	52.1 ± 0.4	52.0 ± 0.3	52.2 ± 0.3	52.3 ± 0.3	52.6 ± 0.6	0.880
TG (mg/dL)	127.8 ± 3.4	131.3 ± 2.7	127.9 ± 3.0	123.9 ± 2.7	115.5 ± 5.6	0.075
Metabolic syndrome (%)	19.4	16.8	14.5	14.1	10.8	<0.001
Obesity phenotype (%)						<0.001
MHNW	61.6	64.9	70.1	70.7	76.0	
MANW	5.7	4.6	4.7	4.5	2.7	
MHO	19.0	18.3	15.3	15.3	13.2	
MAO	13.7	12.2	9.9	9.6	8.1	

Continuous variables are shown as weighted mean ± SE and categorical variables as weighted percentages. *p* values were derived from design-adjusted Wald F tests. ^a^ ≥150 min/week of moderate-intensity or ≥75 min/week of vigorous-intensity physical activity. BMI, body mass index; DBP, diastolic blood pressure; FPG, fasting plasma glucose; HbA1c, glycated hemoglobin; HDL-C, high-density lipoprotein cholesterol; LDL-C, low-density lipoprotein cholesterol; MANW, metabolically abnormal normal weight; MAO, metabolically abnormal obesity; MHNW, metabolically healthy normal weight; MHO, metabolically healthy obesity; SBP, systolic blood pressure; SE, standard error; TG, triglycerides; WC, waist circumference.

**Table 2:** Participant characteristics by obesity phenotype.

	MHNW	MANW	MHO	MAO	*p* Value
n (weighted %)	5306 (68.0)	395 (4.6)	1258 (16.5)	849 (10.9)	
Age (years)	39.4 ± 0.2	51.4 ± 0.8	39.8 ± 0.4	44.4 ± 0.5	<0.001
Male sex (%)	45.3	61.6	61.0	70.7	<0.001
Household income (%)					0.125
Quartile 1 (lowest)	8.8	15.6	9.2	10.1	
Quartile 2	24.1	24.2	24.6	24.8	
Quartile 3	32.4	28.8	32.2	33.9	
Quartile 4 (highest)	34.7	31.4	33.9	31.3	
Education (%)					<0.001
Middle school or less	12.5	32.3	13.8	20.2	
High school	41.2	40.6	42.1	38.6	
College or higher	46.3	27.1	44.1	41.2	
Urban residence (%)	86.0	77.8	84.2	81.2	0.003
Smoking status (%)					<0.001
Never	60.8	41.3	49.5	39.6	
Past	16.9	21.7	19.3	23.5	
Current	22.4	37.0	31.1	36.9	
Alcohol drinking (%)	81.0	77.1	83.7	82.5	0.023
Regular exercise ^a^ (%)	52.1	47.5	58.7	47.8	<0.001
BMI (kg/m^2^)	21.58 ± 0.03	23.18 ± 0.08	27.12 ± 0.06	28.69 ± 0.13	<0.001
WC (cm)	75.0 ± 0.1	82.7 ± 0.3	87.9 ± 0.2	94.4 ± 0.3	<0.001
SBP (mmHg)	109.7 ± 0.2	127.3 ± 1.1	115.2 ± 0.4	125.4 ± 0.6	<0.001
DBP (mmHg)	72.3 ± 0.2	82.0 ± 0.7	76.0 ± 0.3	84.1 ± 0.4	<0.001
FPG (mg/dL)	91.4 ± 0.2	109.1 ± 1.5	94.2 ± 0.3	105.9 ± 0.8	<0.001
HbA1c (%)	5.45 ± 0.01	5.92 ± 0.05	5.56 ± 0.01	5.85 ± 0.03	<0.001
Total cholesterol (mg/dL)	183.0 ± 0.5	196.5 ± 2.1	194.1 ± 1.1	203.6 ± 1.5	<0.001
LDL-C (mg/dL)	108.7 ± 0.5	115.5 ± 2.1	120.5 ± 1.0	123.8 ± 1.3	<0.001
HDL-C (mg/dL)	55.0 ± 0.2	42.5 ± 0.5	50.2 ± 0.3	41.4 ± 0.3	<0.001
TG (mg/dL)	99.9 ± 1.1	237.3 ± 8.4	130.4 ± 3.2	246.7 ± 7.1	<0.001
Sleep duration (h/day)	6.87 ± 0.02	6.64 ± 0.07	6.67 ± 0.04	6.64 ± 0.05	<0.001
Sleep duration category (%)					<0.001
≤5 h	11.5	15.8	14.7	16.1	
6 h	27.5	28.6	32.1	32.5	
7 h	31.3	30.9	28.2	27.6	
8 h	22.4	21.0	19.9	19.0	
≥9 h	7.2	3.7	5.2	4.8	

Continuous variables are shown as weighted mean ± SE and categorical variables as weighted percentages. *p* values were derived from design-adjusted Wald F tests. ^a^ ≥150 min/week of moderate-intensity or ≥75 min/week of vigorous-intensity physical activity. BMI, body mass index; DBP, diastolic blood pressure; FPG, fasting plasma glucose; HbA1c, glycated hemoglobin; HDL-C, high-density lipoprotein cholesterol; LDL-C, low-density lipoprotein cholesterol; MANW, metabolically abnormal normal weight; MAO, metabolically abnormal obesity; MHNW, metabolically healthy normal weight; MHO, metabolically healthy obesity; SBP, systolic blood pressure; SE, standard error; TG, triglycerides; WC, waist circumference.

**Table 3:** Association between sleep duration and obesity phenotypes.

Sleep Duration	MHNW	MANW OR (95% CI)	*p* Value	MHO OR (95% CI)	*p* Value	MAO OR (95% CI)	*p* Value
**Model 1** (n = 7808)							
≤5 h	Reference	1.933 (1.033–3.619)	0.039	1.707 (1.173–2.484)	0.005	1.696 (1.078–2.669)	0.022
6 h	Reference	1.646 (0.933–2.902)	0.085	1.493 (1.057–2.109)	0.023	1.394 (0.911–2.133)	0.126
7 h	Reference	1.683 (0.946–2.996)	0.077	1.184 (0.831–1.686)	0.349	1.099 (0.722–1.672)	0.659
8 h	Reference	1.624 (0.888–2.969)	0.115	1.179 (0.811–1.712)	0.388	1.093 (0.705–1.695)	0.691
≥9 h	Reference	1 (reference)		1 (reference)		1 (reference)	
**Model 2** (n = 7746)							
≤5 h	Reference	2.063 (1.070–3.978)	0.031	1.650 (1.128–2.413)	0.010	1.649 (1.047–2.597)	0.031
6 h	Reference	1.876 (1.033–3.409)	0.039	1.495 (1.049–2.131)	0.026	1.442 (0.940–2.211)	0.093
7 h	Reference	1.870 (1.010–3.460)	0.046	1.195 (0.831–1.719)	0.335	1.120 (0.735–1.708)	0.597
8 h	Reference	1.772 (0.945–3.324)	0.074	1.168 (0.799–1.707)	0.423	1.096 (0.706–1.701)	0.683
≥9 h	Reference	1 (reference)		1 (reference)		1 (reference)	

ORs and 95% CIs were estimated using survey-weighted multinomial logistic regression with MHNW as the reference phenotype (OR fixed at 1) and sleep duration of ≥9 h as the reference category. Model 1 included age and sex; Model 2 additionally included household income, educational attainment, smoking status, alcohol drinking, and regular exercise. CI, confidence interval; MANW, metabolically abnormal normal weight; MAO, metabolically abnormal obesity; MHNW, metabolically healthy normal weight; MHO, metabolically healthy obesity; OR, odds ratio.

## Data Availability

The data used in this study are publicly available from the KNHANES website (https://knhanes.kdca.go.kr).

## Supplementary Materials

The following supporting information can be downloaded at: https://www.mdpi.com/article/10.3390/medicina62102014/s1, Table S1: Association between sleep duration and obesity phenotypes using 7 h/day as the reference sleep duration (sensitivity analysis); Table S2: Association between sleep duration and obesity phenotypes using a stricter definition of metabolic health; Table S3: Association between sleep duration and obesity phenotypes after excluding underweight participants; Table S4: Association between sleep duration and obesity phenotypes with additional adjustment for residential area, perceived stress, total energy intake, and work schedule (Model 3); Table S5: Association between sleep duration and obesity phenotypes stratified by sex and age group; Table S6: Variance inflation factors for the covariates included in the multivariable models.

## References

[1] Jeong S.M., Jung J.H., Yang Y.S., Kim W., Cho I.Y., Lee Y.B., Park K.Y., Nam G.E., Han K. (2024). 2023 Obesity fact sheet: Prevalence of obesity and abdominal obesity in adults, adolescents, and children in Korea from 2012 to 2021. J. Obes. Metab. Syndr..

[2] Rhee E.J. (2025). Prevalence and current status of cardiometabolic risk factors in Korean adults based on fact sheets 2024. Endocrinol. Metab..

[3] Park H.R., Lee S.Y., Moon H.J., Kim J.H., Cho J.W., Cho Y.W., Yun C.H., Han S.H., Chu M.K. (2024). Longitudinal changes in sleep and sleep-related symptoms among Korean adults between 2010 to 2022, including the COVID-19 pandemic period. PLoS ONE.

[4] Cappuccio F.P., Taggart F.M., Kandala N.B., Currie A., Peile E., Stranges S., Miller M.A. (2008). Meta-analysis of short sleep duration and obesity in children and adults. Sleep.

[5] Lange M.G., Neophytou C., Cappuccio F.P., Barber T.M., Johnson S., Chen Y.F. (2024). Sex differences in the association between short sleep duration and obesity: A systematic-review and meta-analysis. Nutr. Metab. Cardiovasc. Dis..

[6] Cappuccio F.P., D’Elia L., Strazzullo P., Miller M.A. (2010). Quantity and quality of sleep and incidence of type 2 diabetes: A systematic review and meta-analysis. Diabetes Care.

[7] Shan Z., Ma H., Xie M., Yan P., Guo Y., Bao W., Rong Y., Jackson C.L., Hu F.B., Liu L. (2015). Sleep duration and risk of type 2 diabetes: A meta-analysis of prospective studies. Diabetes Care.

[8] Cappuccio F.P., Cooper D., D’Elia L., Strazzullo P., Miller M.A. (2011). Sleep duration predicts cardiovascular outcomes: A systematic review and meta-analysis of prospective studies. Eur. Heart J..

[9] Hall M.H., Muldoon M.F., Jennings J.R., Buysse D.J., Flory J.D., Manuck S.B. (2008). Self-reported sleep duration is associated with the metabolic syndrome in midlife adults. Sleep.

[10] Choi K.M., Lee J.S., Park H.S., Baik S.H., Choi D.S., Kim S.M. (2008). Relationship between sleep duration and the metabolic syndrome: Korean National Health and Nutrition Survey 2001. Int. J. Obes..

[11] Che T., Yan C., Tian D., Zhang X., Liu X., Wu Z. (2021). The association between sleep and metabolic syndrome: A systematic review and meta-analysis. Front. Endocrinol..

[12] Lloyd-Jones D.M., Allen N.B., Anderson C.A.M., Black T., Brewer L.C., Foraker R.E., Grandner M.A., Lavretsky H., Perak A.M., Sharma G. (2022). Life’s Essential 8: Updating and enhancing the American Heart Association’s construct of cardiovascular health: A presidential advisory from the American Heart Association. Circulation.

[13] Spiegel K., Tasali E., Penev P., Van Cauter E. (2004). Brief communication: Sleep curtailment in healthy young men is associated with decreased leptin levels, elevated ghrelin levels, and increased hunger and appetite. Ann. Intern. Med..

[14] Taheri S., Lin L., Austin D., Young T., Mignot E. (2004). Short sleep duration is associated with reduced leptin, elevated ghrelin, and increased body mass index. PLoS Med..

[15] Buxton O.M., Pavlova M., Reid E.W., Wang W., Simonson D.C., Adler G.K. (2010). Sleep restriction for 1 week reduces insulin sensitivity in healthy men. Diabetes.

[16] Covassin N., Singh P., McCrady-Spitzer S.K., St Louis E.K., Calvin A.D., Levine J.A., Somers V.K. (2022). Effects of experimental sleep restriction on energy intake, energy expenditure, and visceral obesity. J. Am. Coll. Cardiol..

[17] Tasali E., Wroblewski K., Kahn E., Kilkus J., Schoeller D.A. (2022). Effect of sleep extension on objectively assessed energy intake among adults with overweight in real-life settings: A randomized clinical trial. JAMA Intern. Med..

[18] Stefan N., Häring H.U., Hu F.B., Schulze M.B. (2013). Metabolically healthy obesity: Epidemiology, mechanisms, and clinical implications. Lancet Diabetes Endocrinol..

[19] Blüher M. (2020). Metabolically healthy obesity. Endocr. Rev..

[20] Stefan N., Schulze M.B. (2024). Metabolically healthy obesity: From epidemiology and mechanisms to clinical implications. Nat. Rev. Endocrinol..

[21] Mathew H., Farr O.M., Mantzoros C.S. (2016). Metabolic health and weight: Understanding metabolically unhealthy normal weight or metabolically healthy obese patients. Metabolism.

[22] Iacobini C., Pugliese G., Blasetti Fantauzzi C., Federici M., Menini S. (2019). Metabolically healthy versus metabolically unhealthy obesity. Metabolism.

[23] WHO Expert Consultation (2004). Appropriate body-mass index for Asian populations and its implications for policy and intervention strategies. Lancet.

[24] Ryu J.Y., Lee J.S., Hong H.C., Choi H.Y., Yoo H.J., Seo J.A., Kim S.G., Kim N.H., Baik S.H., Choi D.S. (2015). Association between body size phenotype and sleep duration: Korean National Health and Nutrition Examination Survey V (KNHANES V). Metabolism.

[25] Wang J., Xue D., Shi B., Xia L., Chen W., Liu L., Liu J., Wang H., Ye F. (2022). Sleep duration and metabolic body size phenotypes among Chinese young workers. Front. Public Health.

[26] Oh K., Kim Y., Kweon S., Kim S., Yun S., Park S., Lee Y.K., Kim Y., Park O., Jeong E.K. (2021). Korea National Health and Nutrition Examination Survey, 20th anniversary: Accomplishments and future directions. Epidemiol. Health.

[27] Lee H.A., Kim H.R., Park H., Jung S.Y., Jeon J.P., Park B., Jung K.J., Jee S.H., Shim S.M., Park H. (2025). Data resource profile: The statistics of the Korea National Health and Nutrition Examination Survey (KNHANES) biobank project. J. Korean Med. Sci..

[28] Eum M.-J., Si E. (2025). Association between depression and the short sleep duration on weekdays and weekends in South Korean adults: A cross-sectional study using the 2016 and 2018 Korea National Health and Nutrition Examination Survey. Healthcare.

[29] Lyu Y.S., Yoon Y., Kim J.H., Kim S.Y. (2025). Association between incident chronic kidney disease and body size phenotypes in apparently healthy adults: An observational study using the Korean National Health and Nutrition Examination Survey (2019–2021). Biomedicines.

[30] Bae J.H., Haam J.H., Jeon E., Kang S.Y., Song S., Park C.Y., Kwon H. (2025). Committee of Clinical Practice Guidelines, Korean Society for the Study of Obesity (KSSO). 2024 Clinical practice guidelines for the diagnosis and pharmacologic treatment of overweight and obesity by the Korean Society for the Study of Obesity. J. Obes. Metab. Syndr..

[31] VanderWeele T.J. (2019). Principles of confounder selection. Eur. J. Epidemiol..

[32] Hughes R.A., Heron J., Sterne J.A.C., Tilling K. (2019). Accounting for missing data in statistical analyses: Multiple imputation is not always the answer. Int. J. Epidemiol..

[33] Rothman K.J. (1990). No adjustments are needed for multiple comparisons. Epidemiology.

[34] Spiegel K., Leproult R., Van Cauter E. (1999). Impact of sleep debt on metabolic and endocrine function. Lancet.

[35] Leproult R., Copinschi G., Buxton O., Van Cauter E. (1997). Sleep loss results in an elevation of cortisol levels the next evening. Sleep.

[36] Irwin M.R., Olmstead R., Carroll J.E. (2016). Sleep disturbance, sleep duration, and inflammation: A systematic review and meta-analysis of cohort studies and experimental sleep deprivation. Biol. Psychiatry.

[37] Hairston K.G., Bryer-Ash M., Norris J.M., Haffner S., Bowden D.W., Wagenknecht L.E. (2010). Sleep duration and five-year abdominal fat accumulation in a minority cohort: The IRAS family study. Sleep.

[38] Zembic A., Eckel N., Stefan N., Baudry J., Schulze M.B. (2021). An empirically derived definition of metabolically healthy obesity based on risk of cardiovascular and total mortality. JAMA Netw. Open.

