# Supplementary material for: Association Between Sleep Duration and Obesity Phenotypes in Apparently Healthy Korean Adults: A Cross-Sectional Study Using the Korea National Health and Nutrition Examination Survey 2013–2015
Source: Medicina (Kaunas). 2026 Oct 18;62(10):2014. doi: 10.3390/medicina62102014 (PMC13706694; doi:10.3390/medicina62102014)
Supplement: Supplementary file 1 [file medicina-62-02014-s001.zip › medicina-4623498-supplementary.pdf]

Supplementary Materials

Association between Sleep Duration and Obesity Phenotypes in Apparently Healthy Korean Adults

Table S1. Association between sleep duration and obesity phenotypes using 7 h/day as the reference sleep duration (sensitivity analysis).

Sleep Duration	MANW OR (95% CI)	*P Value*	MHO OR (95% CI)	*P Value*	MAO OR (95% CI)	*P Value*
**Model 1 (n = 7,808)**						
≤5 h	1.149 (0.777–1.699)	0.487	1.442 (1.151–1.806)	0.002	1.544 (1.173–2.031)	0.002
6 h	0.978 (0.710–1.346)	0.890	1.261 (1.057–1.504)	0.010	1.268 (1.028–1.564)	0.026
7 h	1 (reference)		1 (reference)		1 (reference)	
8 h	0.965 (0.701–1.328)	0.826	0.995 (0.812–1.220)	0.965	0.995 (0.776–1.275)	0.966
≥9 h	0.594 (0.334–1.058)	0.077	0.845 (0.593–1.203)	0.349	0.910 (0.598–1.385)	0.659
**Model 2 (n = 7,746)**						
≤5 h	1.104 (0.742–1.641)	0.626	1.380 (1.098–1.734)	0.006	1.472 (1.109–1.953)	0.008
6 h	1.003 (0.729–1.381)	0.984	1.251 (1.048–1.492)	0.013	1.287 (1.047–1.583)	0.017
7 h	1 (reference)		1 (reference)		1 (reference)	
8 h	0.948 (0.688–1.307)	0.744	0.977 (0.795–1.200)	0.822	0.978 (0.761–1.257)	0.862
≥9 h	0.535 (0.289–0.990)	0.046	0.836 (0.582–1.203)	0.335	0.893 (0.585–1.361)	0.597

ORs and 95% CIs were estimated using survey-weighted multinomial logistic regression with MHNW as the reference phenotype. Sleep duration of 7 h was used as the reference category. Model 1 was adjusted for age and sex. Model 2 was further adjusted for household income, educational attainment, smoking status, alcohol drinking, and regular exercise. CI, confidence interval; MANW, metabolically abnormal normal weight; MAO, metabolically abnormal obesity; MHNW, metabolically healthy normal weight; MHO, metabolically healthy obesity; OR, odds ratio.

Table S2. Association between sleep duration and obesity phenotypes using a stricter definition of metabolic health.

(A) Weighted distribution of obesity phenotypes under the stricter definition

Sleep Duration	n	MHNW (%)	MANW (%)	MHO (%)	MAO (%)
≤5 h	1,036	32.8	34.5	6.5	26.2
6 h	2,271	36.8	32.6	6.6	24.0
7 h	2,347	39.6	35.2	5.4	19.8
8 h	1,660	38.9	36.3	5.3	19.6
≥9 h	494	47.2	31.5	6.0	15.3
Total	7,808	38.3	34.4	5.9	21.5

(B) Multinomial logistic regression

Sleep Duration	MANW OR (95% CI)	*P Value*	MHO OR (95% CI)	*P Value*	MAO OR (95% CI)	*P Value*
**Reference ≥9 h, Model 1 (n = 7,808)**						
≤5 h	1.026 (0.759–1.387)	0.867	1.401 (0.812–2.418)	0.225	1.697 (1.169–2.464)	0.006
6 h	0.911 (0.697–1.192)	0.498	1.205 (0.737–1.968)	0.457	1.357 (0.969–1.902)	0.076
7 h	0.994 (0.767–1.288)	0.964	0.961 (0.581–1.588)	0.876	1.135 (0.806–1.599)	0.467
8 h	1.117 (0.848–1.470)	0.431	0.975 (0.572–1.664)	0.927	1.225 (0.849–1.765)	0.277
≥9 h	1 (reference)		1 (reference)		1 (reference)	
**Reference ≥9 h, Model 2 (n = 7,746)**						
≤5 h	1.075 (0.795–1.455)	0.638	1.397 (0.805–2.423)	0.234	1.675 (1.146–2.447)	0.008
6 h	0.995 (0.756–1.310)	0.974	1.247 (0.753–2.065)	0.391	1.433 (1.013–2.028)	0.042
7 h	1.086 (0.833–1.414)	0.543	1.002 (0.596–1.685)	0.994	1.201 (0.846–1.705)	0.306
8 h	1.185 (0.898–1.565)	0.229	0.981 (0.569–1.692)	0.944	1.261 (0.869–1.829)	0.221
≥9 h	1 (reference)		1 (reference)		1 (reference)	

Sleep Duration	MANW OR (95% CI)	*P Value*	MHO OR (95% CI)	*P Value*	MAO OR (95% CI)	*P Value*
**Reference 7 h, Model 1 (n = 7,808)**						
≤5 h	1.032 (0.829–1.284)	0.777	1.458 (0.978–2.175)	0.064	1.495 (1.182–1.890)	0.001
6 h	0.917 (0.777–1.082)	0.305	1.254 (0.942–1.668)	0.120	1.196 (1.003–1.425)	0.046
7 h	1 (reference)		1 (reference)		1 (reference)	
8 h	1.123 (0.943–1.337)	0.191	1.015 (0.714–1.444)	0.933	1.079 (0.882–1.319)	0.461
≥9 h	1.006 (0.776–1.303)	0.964	1.041 (0.630–1.720)	0.876	0.881 (0.626–1.240)	0.467
**Reference 7 h, Model 2 (n = 7,746)**						
≤5 h	0.991 (0.794–1.236)	0.933	1.394 (0.926–2.098)	0.111	1.395 (1.099–1.771)	0.006
6 h	0.917 (0.776–1.084)	0.310	1.244 (0.934–1.658)	0.136	1.194 (1.004–1.420)	0.046
7 h	1 (reference)		1 (reference)		1 (reference)	
8 h	1.092 (0.916–1.302)	0.325	0.979 (0.686–1.396)	0.905	1.050 (0.857–1.287)	0.636
≥9 h	0.921 (0.707–1.200)	0.543	0.998 (0.594–1.678)	0.994	0.833 (0.587–1.182)	0.306

Under the stricter definition, participants were classified as metabolically healthy only if they had none of the four metabolic syndrome components other than waist circumference (elevated triglycerides, low HDL cholesterol, elevated blood pressure, and elevated fasting glucose); BMI cut-off, 25 kg/m². Unweighted numbers: MHNW, 2,850; MANW, 2,851; MHO, 433; MAO, 1,674. ORs and 95% CIs were estimated using survey-weighted multinomial logistic regression with MHNW as the reference phenotype. Model 1 was adjusted for age and sex. Model 2 was further adjusted for household income, educational attainment, smoking status, alcohol drinking, and regular exercise. CI, confidence interval; MANW, metabolically abnormal normal weight; MAO, metabolically abnormal obesity; MHNW, metabolically healthy normal weight; MHO, metabolically healthy obesity; OR, odds ratio.

Table S3. Association between sleep duration and obesity phenotypes after excluding underweight participants (BMI <18.5 kg/m², n = 401).

Sleep Duration	MANW OR (95% CI)	*P Value*	MHO OR (95% CI)	*P Value*	MAO OR (95% CI)	*P Value*
**Reference ≥9 h, Model 1 (n = 7,407)**						
≤5 h	1.839 (0.983–3.442)	0.057	1.544 (1.059–2.251)	0.024	1.563 (0.996–2.452)	0.052
6 h	1.539 (0.872–2.716)	0.137	1.354 (0.959–1.912)	0.085	1.276 (0.834–1.952)	0.261
7 h	1.598 (0.895–2.855)	0.113	1.083 (0.761–1.542)	0.656	1.016 (0.668–1.545)	0.942
8 h	1.530 (0.831–2.817)	0.172	1.107 (0.762–1.608)	0.592	1.030 (0.665–1.595)	0.895
≥9 h	1 (reference)		1 (reference)		1 (reference)	
**Reference ≥9 h, Model 2 (n = 7,346)**						
≤5 h	1.961 (1.017–3.783)	0.044	1.503 (1.027–2.200)	0.036	1.514 (0.964–2.378)	0.071
6 h	1.743 (0.956–3.177)	0.070	1.363 (0.957–1.941)	0.086	1.318 (0.861–2.018)	0.204
7 h	1.769 (0.949–3.295)	0.072	1.103 (0.768–1.584)	0.595	1.034 (0.679–1.575)	0.875
8 h	1.665 (0.880–3.151)	0.117	1.103 (0.755–1.613)	0.612	1.030 (0.664–1.596)	0.896
≥9 h	1 (reference)		1 (reference)		1 (reference)	
**Reference 7 h, Model 1 (n = 7,407)**						
≤5 h	1.151 (0.777–1.704)	0.482	1.425 (1.137–1.786)	0.002	1.538 (1.168–2.026)	0.002
6 h	0.963 (0.699–1.326)	0.816	1.250 (1.048–1.491)	0.013	1.256 (1.018–1.551)	0.034
7 h	1 (reference)		1 (reference)		1 (reference)	
8 h	0.957 (0.691–1.326)	0.792	1.022 (0.833–1.253)	0.834	1.014 (0.790–1.300)	0.914
≥9 h	0.626 (0.350–1.118)	0.113	0.923 (0.649–1.313)	0.656	0.984 (0.647–1.498)	0.942
**Reference 7 h, Model 2 (n = 7,346)**						
≤5 h	1.109 (0.746–1.649)	0.609	1.363 (1.084–1.713)	0.008	1.464 (1.103–1.944)	0.008
6 h	0.985 (0.715–1.358)	0.928	1.235 (1.035–1.474)	0.019	1.274 (1.035–1.568)	0.022
7 h	1 (reference)		1 (reference)		1 (reference)	
8 h	0.941 (0.678–1.306)	0.717	1.000 (0.814–1.228)	0.998	0.995 (0.774–1.280)	0.972
≥9 h	0.565 (0.304–1.053)	0.072	0.907 (0.631–1.302)	0.595	0.967 (0.635–1.472)	0.875

ORs and 95% CIs were estimated using survey-weighted multinomial logistic regression with MHNW as the reference phenotype. Model 1 was adjusted for age and sex. Model 2 was further adjusted for household income, educational attainment, smoking status, alcohol drinking, and regular exercise. BMI, body mass index; CI, confidence interval; MANW, metabolically abnormal normal weight; MAO, metabolically abnormal obesity; MHNW, metabolically healthy normal weight; MHO, metabolically healthy obesity; OR, odds ratio.

Table S4. Association between sleep duration and obesity phenotypes with additional adjustment for residential area, perceived stress, total energy intake, and work schedule (Model 3).

Sleep Duration	MANW OR (95% CI)	*P Value*	MHO OR (95% CI)	*P Value*	MAO OR (95% CI)	*P Value*
**Reference ≥9 h, Model 2 in the Model 3 sample (n = 6,854)**						
≤5 h	2.611 (1.236–5.512)	0.012	1.515 (1.013–2.264)	0.043	1.548 (0.962–2.492)	0.072
6 h	1.952 (0.971–3.924)	0.061	1.339 (0.929–1.930)	0.118	1.353 (0.867–2.111)	0.183
7 h	2.156 (1.062–4.375)	0.033	1.078 (0.745–1.558)	0.691	1.064 (0.688–1.645)	0.780
8 h	2.113 (1.036–4.310)	0.040	1.094 (0.741–1.617)	0.650	0.953 (0.604–1.502)	0.835
≥9 h	1 (reference)		1 (reference)		1 (reference)	
**Reference ≥9 h, Model 3 (n = 6,854)**						
≤5 h	2.693 (1.285–5.645)	0.009	1.495 (1.002–2.231)	0.049	1.511 (0.939–2.431)	0.089
6 h	1.985 (0.984–4.007)	0.056	1.330 (0.922–1.920)	0.127	1.329 (0.852–2.074)	0.210
7 h	2.202 (1.081–4.486)	0.030	1.077 (0.744–1.560)	0.693	1.060 (0.686–1.636)	0.793
8 h	2.141 (1.045–4.385)	0.037	1.099 (0.744–1.624)	0.634	0.945 (0.602–1.484)	0.807
≥9 h	1 (reference)		1 (reference)		1 (reference)	
**Reference 7 h, Model 2 in the Model 3 sample (n = 6,854)**						
≤5 h	1.211 (0.793–1.850)	0.375	1.406 (1.099–1.797)	0.007	1.455 (1.062–1.993)	0.020
6 h	0.905 (0.637–1.287)	0.579	1.243 (1.028–1.502)	0.025	1.271 (1.017–1.590)	0.035
7 h	1 (reference)		1 (reference)		1 (reference)	
8 h	0.980 (0.696–1.380)	0.909	1.016 (0.820–1.258)	0.887	0.895 (0.685–1.170)	0.418
≥9 h	0.464 (0.229–0.941)	0.033	0.928 (0.642–1.341)	0.691	0.940 (0.608–1.453)	0.780
**Reference 7 h, Model 3 (n = 6,854)**						
≤5 h	1.223 (0.802–1.865)	0.350	1.388 (1.086–1.775)	0.009	1.426 (1.043–1.949)	0.026
6 h	0.901 (0.635–1.280)	0.562	1.235 (1.021–1.493)	0.030	1.254 (1.004–1.567)	0.046
7 h	1 (reference)		1 (reference)		1 (reference)	
8 h	0.972 (0.689–1.371)	0.872	1.020 (0.823–1.266)	0.854	0.892 (0.684–1.163)	0.398
≥9 h	0.454 (0.223–0.925)	0.030	0.928 (0.641–1.344)	0.693	0.944 (0.611–1.457)	0.793

ORs and 95% CIs were estimated using survey-weighted multinomial logistic regression with MHNW as the reference phenotype. Model 2 was further adjusted for household income, educational attainment, smoking status, alcohol drinking, and regular exercise. Model 3 was further adjusted for residential area (urban/rural), perceived stress (high/low), total energy intake (kcal/day, continuous), and work schedule (day work, non-day work [evening, night, or shift work], or not working). Model 3 included participants with complete data on all covariates (missing total energy intake, n = 895; perceived stress, n = 5; work schedule, n = 21); Model 2 was refitted in the same participants for comparison. CI, confidence interval; MANW, metabolically abnormal normal weight; MAO, metabolically abnormal obesity; MHNW, metabolically healthy normal weight; MHO, metabolically healthy obesity; OR, odds ratio.

Table S5. Association between sleep duration and obesity phenotypes stratified by sex and age group (reference sleep duration, 7 h; Model 2).

Sleep Duration	MANW OR (95% CI)	*P Value*	MHO OR (95% CI)	*P Value*	MAO OR (95% CI)	*P Value*
**Men (n = 3,389)**						
≤5 h	0.865 (0.472–1.584)	0.638	1.390 (0.991–1.949)	0.056	1.473 (1.008–2.151)	0.045
6 h	1.195 (0.785–1.820)	0.406	1.239 (0.966–1.590)	0.092	1.414 (1.092–1.830)	0.009
7 h	1 (reference)		1 (reference)		1 (reference)	
8 h	0.946 (0.613–1.460)	0.802	0.981 (0.729–1.320)	0.900	1.002 (0.723–1.390)	0.988
≥9 h	0.297 (0.125–0.707)	0.006	0.878 (0.508–1.518)	0.641	0.999 (0.571–1.748)	0.998

Sleep Duration	MANW OR (95% CI)	*P Value*	MHO OR (95% CI)	*P Value*	MAO OR (95% CI)	*P Value*
**Women (n = 4,357)**						
≤5 h	1.272 (0.758–2.137)	0.362	1.233 (0.911–1.670)	0.174	1.269 (0.829–1.943)	0.273
6 h	0.674 (0.417–1.089)	0.107	1.195 (0.908–1.573)	0.204	0.966 (0.656–1.421)	0.859
7 h	1 (reference)		1 (reference)		1 (reference)	
8 h	1.036 (0.643–1.669)	0.884	1.018 (0.772–1.341)	0.901	1.002 (0.685–1.466)	0.992
≥9 h	0.934 (0.411–2.123)	0.870	0.874 (0.557–1.372)	0.558	0.836 (0.441–1.586)	0.584
**Age 20–39 years (n = 3,278)**						
≤5 h	1.587 (0.663–3.799)	0.299	1.261 (0.853–1.865)	0.244	1.641 (0.979–2.749)	0.060
6 h	1.071 (0.476–2.409)	0.868	1.260 (0.971–1.635)	0.082	1.283 (0.880–1.869)	0.195
7 h	1 (reference)		1 (reference)		1 (reference)	
8 h	1.144 (0.442–2.960)	0.781	1.187 (0.881–1.599)	0.260	1.255 (0.824–1.910)	0.289
≥9 h	0.501 (0.062–4.078)	0.518	0.886 (0.538–1.460)	0.635	1.418 (0.748–2.687)	0.284
**Age ≥40 years (n = 4,468)**						
≤5 h	1.079 (0.702–1.658)	0.729	1.454 (1.071–1.974)	0.017	1.447 (1.044–2.005)	0.026
6 h	0.985 (0.695–1.397)	0.933	1.197 (0.940–1.523)	0.144	1.265 (0.978–1.635)	0.073
7 h	1 (reference)		1 (reference)		1 (reference)	
8 h	0.914 (0.631–1.324)	0.634	0.805 (0.609–1.065)	0.129	0.903 (0.665–1.227)	0.513
≥9 h	0.648 (0.348–1.206)	0.171	1.050 (0.627–1.757)	0.853	0.885 (0.505–1.550)	0.669

ORs and 95% CIs were estimated using survey-weighted multinomial logistic regression with MHNW as the reference phenotype. Sleep duration of 7 h was used as the reference category because of the small number of participants sleeping ≥9 h within strata. Models were adjusted for age, sex (age-group strata only), household income, educational attainment, smoking status, alcohol drinking, and regular exercise. P for interaction (design-adjusted Wald F test of sleep duration × sex or age-group terms in the total sample): sex, overall 0.119 (MANW 0.006, MHO 0.983, MAO 0.547); age group, overall 0.870 (MANW 0.976, MHO 0.230, MAO 0.890). Phenotype counts (MHNW/MANW/MHO/MAO): Men, 2014/207/646/522; Women, 3251/181/605/320; Age 20–39 y, 2415/46/559/258; Age ≥40 y, 2850/342/692/584. CI, confidence interval; MANW, metabolically abnormal normal weight; MAO, metabolically abnormal obesity; MHNW, metabolically healthy normal weight; MHO, metabolically healthy obesity; OR, odds ratio.

Table S6. Variance inflation factors for the covariates included in the multivariable models.

Variable	Model 2 Max VIF	Model 2 GVIF^(1/2df)	Model 3 Max VIF	Model 3 GVIF^(1/2df)
Sleep duration	4.13	1.01	4.18	1.01
Age	1.56	1.25	1.63	1.27
Sex	1.79	1.34	1.98	1.41
Household income	3.16	1.03	3.24	1.04
Educational attainment	2.91	1.13	2.97	1.14
Smoking status	1.68	1.16	1.68	1.17
Alcohol drinking	1.12	1.06	1.12	1.06
Regular exercise	1.04	1.02	1.04	1.02
Residential area	–	–	1.06	1.03
Perceived stress	–	–	1.05	1.02
Total energy intake	–	–	1.19	1.09
Work schedule	–	–	1.18	1.04

VIFs were calculated for each indicator variable from linear regressions of each covariate on all other covariates; for categorical variables, the maximum VIF across indicator variables is shown. GVIF^(1/2df), generalized variance inflation factor adjusted for the degrees of freedom of each variable (values $<\sqrt{5} \approx 2.24$ indicate no meaningful multicollinearity). VIF, variance inflation factor.
